# When Standards Protect and Pressure Undermines: A Bayesian and Person-Centred Analysis of Perfectionism, Self-Handicapping, Motivation, and Academic Well-Being in Gifted Adolescents

**DOI:** 10.3390/bs16101904

**Published:** 2026-10-15

**Authors:** Halil Aslan

**Affiliations:** Department of Educational Sciences, Siirt University, 56100 Siirt, Türkiye; halil.aslan@siirt.edu.tr

**Keywords:** perfectionism, self-handicapping, academic motivation, academic well-being, parental achievement pressure, gifted adolescents

## Abstract

Perfectionism is common among gifted students, but how its dimensions relate to academic well-being remains unclear. In this cross-sectional, single-site study, 234 adolescents attending Science and Art Centres in Turkiye (aged 14 to 18; 122 girls, 112 boys) completed measures of perfectionistic standards and discrepancy, academic self-handicapping, academic motivation, subjective academic well-being, and parental achievement pressure. Since academic motivation and subjective academic well-being were not empirically separable in this sample (latent r = 0.96), the two were modelled as a single academic well-being construct, and the originally planned serial model was replaced by a simple indirect model. Discrepancy was not associated with self-handicapping or with academic well-being, and a Bayesian region-of-practical-equivalence analysis placed 99.9% of the posterior for its indirect association within a negligible-effect region. Higher perfectionistic standards were associated with less self-handicapping and, through it, with higher academic well-being; parental achievement pressure showed the reverse pattern. Both indirect associations were statistically reliable but small, and most of their posterior mass also fell within the negligible region. No evidence of moderation by parental pressure was found. Latent profile analysis supported a two-profile solution differing only in standards. Because the data are cross-sectional and single-site, all findings are reported as concurrent associations rather than as processes.

## 1. Introduction

High ability is no guarantee that a student will feel good about school. Some gifted adolescents flourish academically and personally, while others withdraw from challenge, coast well below their measured capacity, or quietly report that their academic lives bring them little satisfaction. Systematic reviews of underachievement in this population describe a tangle of internal and external contributors rather than a single deficit (Raoof et al., 2024), and comparative work on subjective well-being in gifted samples has produced results inconsistent enough to caution against assuming that this group is either uniformly advantaged or uniformly at risk (Casino-Garcia et al., 2019; Kara & Aslan, 2025). The practical question for schools is therefore not whether gifted students differ on average from their peers, but which students within a selective setting are faring poorly and what is associated with that.

Two framing points are needed before proceeding. First, gifted and high-achieving are not interchangeable terms, and we use them separately throughout. Second, the international literature does not support a blanket assumption that gifted students are more perfectionistic than non-gifted students; the evidence is mixed, and our design cannot adjudicate the question because it includes no comparison group.

Perfectionism nonetheless sits close to the heart of the variability that concerns us. Perfectionistic striving is common in academically selective environments, and cohort analyses indicate that perfectionism has been rising among young people over recent decades (Curran & Hill, 2019). In gifted samples specifically, performance perfectionism has been linked to school stress and, through it, to higher burnout and lower engagement (Grugan et al., 2025), and perfectionism has been associated with stress and disrupted self-regulation during periods of disrupted schooling (Hill & Madigan, 2022). These studies establish that perfectionism matters in this population; they leave open the behavioural route by which it does so.

The Almost Perfect Scale—Revised separates standards, the pursuit of high personal performance expectations, from discrepancy, the persistent sense of falling short of those expectations (Rice et al., 2014). Rice and Richardson (2014) have shown that the two behave differently enough that classifying people as perfectionists on the basis of standards alone produces unstable groupings. Standards tend to accompany engagement and achievement, whereas discrepancy is the dimension that travels with distress: meta-analytic work links perfectionistic concerns to burnout (Hill & Curran, 2016) and, more seriously, to suicidality (Smith et al., 2018). The pattern extends to well-being specifically. Among emerging adults, discrepancy was the strongest single correlate of low psychological well-being, while high standards carried a positive sign (Benedetto et al., 2024), and in a Turkish gifted sample positive perfectionism was associated with higher life satisfaction, while negative perfectionism rose across grade levels (Findik & Afat, 2023).

A terminological clarification follows. We refer to the second dimension as discrepancy rather than as maladaptive perfectionism. In the APS-R tradition, a maladaptive perfectionist profile is defined by the conjunction of high standards and high discrepancy, not by discrepancy in isolation, and conflating the dimension with the profile invites exactly the overinterpretation we are trying to avoid. We also flag in advance a possibility that becomes important later: discrepancy may behave differently in academically selective settings, where a felt gap between aspiration and attainment may be so widely shared that it ceases to differentiate students.

If perfectionism reaches academic outcomes through what students actually do, academic self-handicapping is a natural candidate. Self-handicapping is the practice of creating or advertising an obstacle to one’s own performance so that a subsequent failure can be attributed to the obstacle rather than to ability (Urdan & Midgley, 2001). The behaviour is self-protective by design: it trades the prospect of success for insurance against a damaging attribution. Students who carry a chronic sense of falling short have an obvious motive for such insurance, which is why discrepancy has been the dimension most often expected to predict it. Experimental and correlational work indicates that the strategy carries a real cost, since handicapping undermines the effortful, self-regulated engagement on which later performance depends (McCrea, 2008; Thomas & Gadbois, 2006).

Importantly, the two dimensions appear to pull in opposite directions here. Among elite athletes, perfectionistic concerns predicted more self-handicapping, whereas striving predicted less, with attributional style partially accounting for the association (Torok et al., 2022). Self-handicapping also belongs to a broader family of self-undermining behaviours through which students degrade their own study conditions and thereby their well-being (Bakker & Mostert, 2024).

The outcome side of this chain has usually been specified in motivational terms. Self-determined motivation is among the most consistent correlates of academic well-being, and meta-analytic evidence identifies the antecedents that support it (Bureau et al., 2022; Vansteenkiste & Ryan, 2013). It was on this basis that we originally positioned academic motivation between self-handicapping and subjective academic well-being.

As will become clear in the Section 3, this specification did not survive an explicit test of discriminant validity. We retain the rationale here because it motivated the design, but we alert the reader that motivation and well-being proved empirically inseparable in this sample and that the analysis was restructured accordingly.

Perfectionism is also cultivated rather than merely carried, and for high-achievers the family is where much of that cultivation happens. Parental achievement pressure describes a pattern in which parents remain unsatisfied with success, measure the child against high-achieving peers, express blame when results disappoint, and make approval contingent on attainment. Haspolat and Yalcin (2022) developed the construct specifically for high-achieving students, distinguishing it from ordinary parental involvement. Qualitative work in gifted settings describes this blend of competition and expectation as a strain on students’ social and emotional lives (Noor, 2023), and profile research among junior athletes shows that a pressuring parental climate combined with perfectionism is associated with burnout (Gustafsson et al., 2016). The role such pressure plays is open: it may operate as an antecedent that increases self-protective behaviour, or as a moderator that intensifies the cost of that behaviour. The two readings imply different intervention targets, and both are testable in the same model.

A final consideration concerns the level at which perfectionism is examined. Variable-centred analyses describe average associations but say nothing about the configurations of standards and discrepancy that occur within individuals. Person-centred work has identified perfectionistic profiles among school students and shown them to be moderately stable and differentially related to achievement goals and well-being (Kuusi et al., 2024; Stahlberg et al., 2021). Whether comparable configurations emerge in an academically selective sample, and whether they add anything beyond the two dimensions considered jointly, has not been established.

Three contributions follow. First, the study separates the two APS-R dimensions inside a single model of academic well-being in a gifted sample. Second, it subjects a widely assumed motivational pathway to an explicit discriminant-validity test and reports what follows when that test fails, which we believe is informative for a literature that routinely models motivation and academic well-being as distinct. Third, it pairs Bayesian estimation, which permits evidence for practically negligible effects to be quantified rather than inferred from a non-significant interval, with a person-centred analysis in which theoretical expectations are deliberately excluded from class enumeration.

We tested the following. Discrepancy would be positively associated with academic self-handicapping (Hypothesis 1); self-handicapping would be negatively associated with academic motivation (Hypothesis 2); and motivation would be positively associated with subjective academic well-being (Hypothesis 3). The association between discrepancy and well-being would be serially transmitted through higher self-handicapping and lower motivation (Hypothesis 4), and parental achievement pressure would moderate the self-handicapping to motivation association (Hypothesis 5). As secondary questions, we entered the two perfectionism dimensions together and examined whether parental pressure functioned as an antecedent rather than only as a moderator. The latent profile analysis was exploratory.

Hypotheses 2 to 4 presuppose that motivation and academic well-being are distinct. Because that presupposition failed empirically, these hypotheses could not be tested as stated; Section 3.2 reports the test and Section 2.3 describes the model we substituted.

## 2. Method

### 2.1. Participants and Procedure

The participants were 234 adolescents (122 girls and 112 boys) attending Elazığ and Siirt Science and Art in Turkiye. In the Turkish system, Science and Art Centres (Bilim ve Sanat Merkezleri) are publicly funded institutions enrolling students identified through a staged national procedure comprising teacher nomination, a group screening instrument and an individual intelligence assessment, with separate identification pathways for general intellectual ability, visual arts and music. In the present sample, students were identified for general intellectual ability. Of the 600 eligible students, 234 took part, a participation rate of 39%. Ages ranged from 14 to 18 years (*M* = 15.29, *SD* = 0.98). Ninth-graders made up the largest group (*n* = 133), followed by tenth- (*n* = 70) and eleventh-graders (*n* = 29); one twelfth-grader and one case with an unusable grade code, which we set to missing, completed the roster. Students answered a paper questionnaire during regular school hours once institutional permission had been granted. A grade-point-average item was included but went unanswered by 125 students (53.4%). Before setting it aside, we tested whether its missingness was systematic: students with and without a GPA response did not differ on any study variable (all *p* > 0.23, |*d*| ≤ 0.16) or by gender, chi-square(1) = 1.95, *p* = 0.162, and differed only marginally in age, *p* = 0.017, *d* = −0.31. Item-level missingness on the scales was negligible (23 of 12,870 responses, 0.18%, across 20 students). Scale scores were computed as the means of available items provided at least 80% of a scale’s items were answered; no participant fell below this threshold, so the composite dataset was complete. Ethical approval was obtained from the Siirt University Institutional Review Board (protocol number: 9258).

### 2.2. Measures

Subjective academic well-being. Subjective well-being was measured with the six-item Subjective Academic Wellbeing Measure (Arslan et al., 2022). Items use a five-point scale, and higher scores mark greater well-being. Internal consistency in this sample was good (omega = 0.85, alpha = 0.83).

Academic motivation. Motivation was assessed with the 20-item Academic Motivation Scale (Bozanoglu, 2004), answered on a five-point scale with one reverse-scored item. Higher scores indicate stronger motivation, and reliability was excellent (omega = 0.93, alpha = 0.93).

Academic self-handicapping. Self-handicapping was measured with a six-item academic self-handicapping scale in its Turkish form (Anlı et al., 2018), answered on a five-point scale, where higher scores signal heavier use of self-handicapping strategies. The original form was developed by Urdan and Midgley (2001). Reliability was good (omega = 0.81, alpha = 0.81).

Perfectionism. Standards and discrepancy were measured with the adolescent Turkish short form of the Almost Perfect Scale—Revised (Kokcam & Tras, 2026; original short form by Rice et al., 2014). The measure uses a seven-point scale and yields a standards subscale (items 1, 3, 5, and 7) and a discrepancy subscale (items 2, 4, 6, and 8). Reliability was good for standards (omega = 0.84, alpha = 0.84) and modest for discrepancy (omega = 0.64, alpha = 0.62); the weaker figure traces to a single low-loading item that we retained to stay faithful to the published scale.

Parental achievement pressure. Perceived pressure was measured with the 15-item Parental Achievement Pressure Scale, developed for high-achieving students (Haspolat & Yalcin, 2022) and answered on a five-point scale, with higher scores meaning more perceived pressure. Reliability was excellent (omega = 0.93, alpha = 0.93).

### 2.3. Analytic Strategy

All measurement models were estimated with diagonally weighted least squares with robust corrections (WLSMV), treating five-point items as ordinal, following evidence that treating ordinal indicators as continuous distorts fit and parameter estimates when categories are few (Flora & Curran, 2004; Rhemtulla et al., 2012). This materially changed fit relative to the maximum likelihood solution reported in the submitted version. The seven-point APS-R items were kept continuous because one response category was unused. We report at least two indices from different families, interpreted against Hu and Bentler (1999).

Because a discriminant-validity test (Section 3.2) showed that motivation and well-being were not separable, the serial model was not retained. The two were combined into a single latent academic well-being construct, and the structural model was reduced to a simple indirect model with self-handicapping as the intervening variable. Multi-item constructs were represented by parcels built through item-to-construct balancing (Little et al., 2013); because parcelling can conceal multidimensionality, the entire model was re-estimated at the item level as a sensitivity analysis. Indirect associations were tested with Monte Carlo confidence intervals (Preacher, 2015).

The model was re-estimated in a Bayesian framework using blavaan with a Stan backend (Merkle & Rosseel, 2018; Merkle et al., 2021), with package default weakly informative priors, two chains and 1000 post-warmup draws per chain. The Bayesian component was not intended to reproduce the frequentist estimates. Its purpose was to quantify evidence for practically negligible effects, which a non-significant interval cannot do. We defined a region of practical equivalence of |beta| < 0.05 for standardised indirect associations and report the proportion of the posterior falling inside it (Kruschke, 2018). Prior sensitivity compared the defaults against Normal(0, 1) and Normal(0, 100) alternatives, chosen to bracket a plausibly informative and an essentially uninformative specification.

For the person-centred analysis, we compared one- to five-profile solutions across four variance and covariance parameterisations, reporting log-likelihood, AIC, BIC, sample-size-adjusted BIC, entropy and class sizes, plus a parametric bootstrapped likelihood ratio test. Enumeration rested on BIC and the BLRT, which simulation work favours (Nylund-Gibson & Choi, 2018); theoretical expectations were excluded from the criteria. Profiles were compared on distal outcomes using BCH weights, which correct for classification error rather than treating modal assignment as known (Asparouhov & Muthen, 2014; Bolck et al., 2004), with Holm correction across outcomes. Power for the indirect associations was evaluated by Monte Carlo simulation (300 replications) at *N* = 234.

Because the design is cross-sectional and single-site, all results are described as concurrent associations. Cross-sectional indirect effects can diverge substantially from their longitudinal counterparts and can appear when the longitudinal effect is absent (Maxwell & Cole, 2007; Maxwell et al., 2011); direct and indirect effects are retained only as conventional decomposition labels.

### 2.4. Assumption Checks and Software

Univariate distributions were acceptable (|skew| ≤ 0.77, |kurtosis| ≤ 1.11). Multivariate normality was not met (Mardia, *p* < 0.01), variance inflation factors stayed at or below 1.96, no multivariate outliers emerged (Mahalanobis, *p* < 0.001), and Durbin–Watson statistics ranged from 1.75 to 1.94. Homoscedasticity held for two out of three equations but not for the well-being equation (Breusch-Pagan *p* = 0.002), and RESET indicated slight departures from linearity. Kaiser–Meyer–Olkin values ranged from 0.74 to 0.91 with significant Bartlett tests.

Analyses used R 4.5.2 (R Core Team, 2025) with lavaan (Rosseel, 2012), semTools (Jorgensen et al., 2012), blavaan (Merkle & Rosseel, 2018; Merkle et al., 2021) via rstan (Guo et al., 2015), mclust (Scrucca et al., 2023), tidyLPA (Rosenberg et al., 2018), psych (Revelle, 2007), naniar (Tierney & Cook, 2023), MVN (Korkmaz et al., 2014), car (Fox & Weisberg, 2019), lmtest (Hothorn et al., 1999) and ggplot2 (Wickham, 2016).

## 3. Results

### 3.1. Descriptive Statistics and Bivariate Associations

Mean levels were consistent with an academically selective sample. Perfectionistic standards were high (*M* = 5.32, *SD* = 1.30 on a seven-point scale), discrepancy was moderate (*M* = 3.72, *SD* = 1.30), and self-handicapping was low (*M* = 2.46, *SD* = 0.98 on a five-point scale). Subjective academic well-being (*M* = 3.89, *SD* = 0.79) and academic motivation (*M* = 3.45, *SD* = 0.79) were above the scale midpoint, while perceived parental achievement pressure was moderate (*M* = 2.68, *SD* = 1.07). All distributions were within conventional limits for maximum likelihood estimation, with absolute skewness at or below 0.77 and absolute kurtosis at or below 1.11.

The correlation matrix already anticipated two of the paper’s main findings. First, subjective academic well-being was very strongly associated with academic motivation (*r* = 0.83), a magnitude that motivated the discriminant-validity analysis reported below, and strongly associated with perfectionistic standards (*r* = 0.68). Well-being was negatively associated with self-handicapping (*r* = −0.45) and with parental pressure (*r* = −0.27). Second, discrepancy stood apart from the entire network: its correlations with well-being (*r* = 0.03), motivation (*r* = 0.07), self-handicapping (*r* = 0.05) and parental pressure (*r* = 0.02) were all negligible and none reached significance. Its only non-trivial association was a weak positive one with standards (*r* = 0.12, *p* = 0.074), consistent with the modest factor correlation reported below. Self-handicapping and parental pressure were positively associated (*r* = 0.31), and both were negatively associated with standards. Descriptive statistics, internal consistencies and correlations among the study variables are shown in Table 1.

### 3.2. Measurement Models

Estimating the measurement models with an estimator appropriate to ordinal indicators changed the picture reported in the submitted version substantially. Under WLSMV (indicated in Table 2), fit was good for subjective academic well-being (CFI = 0.988, TLI = 0.980, SRMR = 0.044) and acceptable on the incremental indices for self-handicapping (CFI = 0.949, TLI = 0.915, SRMR = 0.063), parental achievement pressure (CFI = 0.945, TLI = 0.936, SRMR = 0.076) and academic motivation (CFI = 0.941, TLI = 0.934, SRMR = 0.065). By comparison, the maximum likelihood solutions reported previously yielded CFI values of 0.946, 0.862, 0.821 and 0.801 for the same four scales. RMSEA remained elevated for the two six-item scales, where the index is known to be upwardly biased at low degrees of freedom, and we therefore weight the incremental indices and SRMR more heavily for those models. We no longer describe the measures as having behaved as intended: four out of five measurement models reached conventional benchmarks on the incremental indices once the ordinal nature of the items was respected, and one did not.

That exception is the APS-R. In its two-dimensional form, the model fitted poorly (CFI = 0.865, TLI = 0.801, RMSEA = 0.205, SRMR = 0.111), and the single-factor alternative was worse still (CFI = 0.711, RMSEA = 0.293), which at least confirms that the two dimensions should not be collapsed. Standardised loadings on standards were uniform (0.71 to 0.80), whereas discrepancy loadings ranged widely (0.28 to 0.81), and the factor correlation was 0.30. Combined with the subscale’s modest reliability (omega = 0.64, alpha = 0.62), this means that the discrepancy dimension is the weakest measured construct in the study. We retain the published scoring for comparability with prior work but treat every discrepancy finding as provisional.

Academic motivation did not reproduce the three-factor structure of the original instrument. Parallel analysis and the minimum average partial criterion both indicated two factors; in a three-factor solution, two items loaded on no factor at the 0.35 threshold and two others cross-loaded. The two-factor solution assigned nine items to one factor and eleven to the other, with a factor correlation of 0.84 in the exploratory solution and 0.84 in the confirmatory model. Two-factor fit exceeded one-factor fit under both estimators (WLSMV: CFI = 0.941 versus 0.914; MLR: CFI = 0.852 versus 0.807), so motivation was modelled with two factors wherever it was retained.

Gender invariance was re-examined on the revised measurement model. Relative to the configural solution, constraining loadings changed fit by dCFI = −0.008 and dRMSEA = 0.000, and additionally constraining intercepts changed fit by dCFI = −0.007 and dRMSEA = 0.000, both within the guidelines of Chen (2007). We note, however, that the configural baseline did not itself reach conventional benchmarks (CFI = 0.860, TLI = 0.828, RMSEA = 0.119, SRMR = 0.090). Because small changes in fit are difficult to interpret when the baseline model does not represent the data well, we report invariance as tentative rather than established and do not use it to license latent mean comparisons.

### 3.3. Discriminant Validity of Motivation and Academic Well-Being

Given a manifest correlation of 0.83 between academic motivation and subjective academic well-being, we tested directly whether the two are separable. Three nested measurement models shown in Table 3 were compared at the item level under WLSMV. A single-factor model merging all 26 items fitted CFI = 0.923, TLI = 0.916, RMSEA = 0.087. A two-factor model separating motivation from well-being fitted marginally better (CFI = 0.924, TLI = 0.917), and the improvement was statistically reliable under maximum likelihood estimation, scaled chi-square difference = 17.56, df = 1, *p* < 0.001. Statistical separability, however, is not the same as empirical distinctness: the latent correlation between the two factors was 0.955.

We then asked whether the problem was an underspecified motivation factor. Modelling motivation with the two factors, the data supported improved overall fit (CFI = 0.937, TLI = 0.931, RMSEA = 0.079) but did not restore discriminant validity: the well-being factor correlated 0.940 with the first motivation factor and 0.901 with the second, while the two motivation factors correlated 0.839 with each other. In other words, well-being was as strongly related to each motivation factor as those factors were to one another. A latent correlation in the mid-90s leaves too little unique variance for the two constructs to occupy distinct positions in a mediational chain.

We therefore abandoned the serial model, combined motivation and well-being into a single academic well-being construct, and proceeded with a simple indirect model. This is a substantive departure from the submitted version and, in our view, the only defensible reading of the measurement evidence. Hypotheses 2 to 4, which presupposed the distinction, could not be tested as stated.

### 3.4. Structural Model

The revised model, in which self-handicapping intervenes between the two perfectionism dimensions and parental pressure on one side and academic well-being on the other, fitted better than the serial model it replaces: CFI = 0.936, TLI = 0.921, RMSEA = 0.077 with SRMR = 0.074, against CFI = 0.893, TLI = 0.870 and RMSEA = 0.093 previously. The improvement is worth noting because the revised model is also more parsimonious.

Turning to the coefficients, discrepancy was unrelated to either endogenous variable. Its association with self-handicapping was near zero (*b* = 0.056, SE = 0.086, *p* = 0.511, beta = 0.068), and its association with academic well-being was indistinguishable from zero (*b* = −0.005, SE = 0.058, *p* = 0.930, beta = −0.007). Perfectionistic standards behaved in the opposite way on both counts: students reporting higher standards reported less self-handicapping (*b* = −0.225, SE = 0.069, *p* = 0.001, beta = −0.295) and higher academic well-being (*b* = 0.505, SE = 0.072, *p* < 0.001, beta = 0.702). Perceived parental achievement pressure was associated with more self-handicapping (*b* = 0.212, SE = 0.059, *p* < 0.001, beta = 0.275) but showed no direct association with well-being once self-handicapping was in the model (*b* = 0.022, SE = 0.039, *p* = 0.575, beta = 0.030), which is consistent with its association operating through the intervening variable. Self-handicapping itself was negatively associated with academic well-being (*b* = −0.233, SE = 0.060, *p* < 0.001, beta = −0.248).

Because parcelling can conceal multidimensionality, the entire model was re-estimated at the item level without parcels under WLSMV (CFI = 0.881, TLI = 0.876, RMSEA = 0.061). Every conclusion was reproduced. Discrepancy remained unrelated to self-handicapping (*p* = 0.212) and to well-being (*p* = 0.245); standards remained associated with less self-handicapping (beta = −0.301) and higher well-being (beta = 0.664); parental pressure remained associated with more self-handicapping (beta = 0.324); and self-handicapping remained negatively associated with well-being (beta = −0.292). The correspondence between the parcelled and item-level solutions indicates that the findings are not an artefact of the parcelling strategy. Standardised structural coefficients for parcelled and item-level models are shown in Table 4.

The indirect associations followed from these coefficients. The association running from standards through lower self-handicapping to higher academic well-being was positive and its interval excluded zero (estimate = 0.052, 95% Monte Carlo CI = [0.016, 0.102]). The corresponding association for parental pressure was negative and also excluded zero (estimate = −0.049, 95% CI = [−0.097, −0.015]). The association for discrepancy was small and straddled zero (estimate = −0.013, 95% CI = [−0.051, 0.030]). In the item-level model, the same three associations were −0.036 (*p* = 0.237), 0.045 (*p* = 0.003) and −0.077 (*p* = 0.002).

A Monte Carlo simulation drawing 300 samples of *N* = 234 from the fitted model returned a power of 0.977 for each of the two non-zero indirect associations. The null result for discrepancy is therefore unlikely to reflect insufficient power for effects of the magnitude observed elsewhere in the same model, although it remains possible that a genuinely smaller association went undetected. Indirect associations with Monte Carlo intervals and ROPE results are presented in Table 5.

### 3.5. Bayesian Estimation, Practical Equivalence, and the Moderation Test

The Bayesian re-estimation converged without difficulty. All potential scale reduction factors fell between 0.999 and 1.001, the smallest effective sample size exceeded 2400, and the posterior predictive *p* value was 0.49, indicating close correspondence between model and data. Posterior means reproduced the maximum likelihood estimates to within rounding.

Prior sensitivity was negligible. Under the package defaults, the posterior mean for the discrepancy pathway was −0.011, 95% credible interval = [−0.032, 0.007]; under an informative Normal(0, 1) prior it was −0.011 [−0.032, 0.008]; and under a diffuse Normal(0, 100) prior it was −0.011 [−0.032, 0.008]. The corresponding values for standards were 0.036 [0.015, 0.062], 0.036 [0.014, 0.061] and 0.036 [0.015, 0.063], and for parental pressure they were −0.040 [−0.073, −0.016], −0.040 [−0.069, −0.017] and −0.040 [−0.069, −0.017]. No substantive conclusion depended on the prior specification.

The region-of-practical-equivalence analysis was the purpose of the Bayesian component and produced the most informative result. For the discrepancy pathway, 99.9% of the posterior fell inside the region defined in advance as practically negligible, which constitutes positive evidence that this association is negligible in this sample rather than merely unproven. The same analysis, however, disciplines our positive findings. For the standards pathway, 86.2% of the posterior also fell inside the negligible region, and for parental pressure 78.5% did. Both associations are therefore reliably non-zero and, at the same time, small enough that most plausible parameter values lie within a range we would not regard as practically meaningful. We report them accordingly.

The moderation hypothesis was not supported. The interaction of parental pressure with self-handicapping in predicting motivation was small and its interval included zero (*b* = −0.04, 95% CI = [−0.14, 0.10]), and the index of moderated mediation included zero for every predictor. We state only that no evidence of moderation was found. Because interaction terms require considerably more power than main effects, and because we did not prespecify a smallest interaction effect of interest against which an equivalence test could be conducted, these data cannot establish that parental pressure does not moderate the association.

### 3.6. Person-Centred Analysis

Enumeration was repeated across one to five profiles and four variance and covariance parameterisations, with theoretical expectations deliberately excluded from the selection criteria. Model fit is reported in full in Table 6. Among the estimable solutions, BIC was lowest for a two-profile model with freely estimated variances and zero covariances (BIC = 1301.95), followed by the corresponding unconstrained two-profile model (1304.20) and the three-profile solution of the same family (1313.82). AIC and the sample-size-adjusted BIC favoured five-profile solutions, as those indices commonly do, and entropy did not discriminate cleanly among candidates. A parametric bootstrapped likelihood ratio test comparing two and three profiles was borderline (2 × difference in log-likelihood = 15.41, *p* = 0.050 across 199 replications).

Weighing BIC and the BLRT over AIC, and noting that the three-profile solution reported in the submitted version had been selected partly because it matched theoretical expectation, we retain two profiles. Classification quality was acceptable, with average posterior probabilities of 0.845 and 0.962 for the two profiles.

The two profiles differed almost entirely on one dimension. Profile 1 (*n* = 85, 36.3%) was characterised by high standards (*M* = 6.63, *z* = +1.01) with discrepancy at the sample average (*M* = 3.76, *z* = +0.03). Profile 2 (*n* = 149, 63.7%) reported markedly lower standards (*M* = 4.57, *z* = −0.58) and, again, average discrepancy (*M* = 3.70, z = −0.02). The two groups were statistically indistinguishable on discrepancy (*z* = 0.36, *p* = 0.715, *d* = 0.06) while differing very substantially on standards (*z* = 22.63, *p* < 0.001, *d* = 1.89). The analysis therefore did not recover a typology crossing the two dimensions; it recovered the standards dimension.

Distal outcomes were compared using BCH weights, which correct for classification error rather than treating modal assignment as certain, with Holm correction across the four outcomes. Students in the high-standards profile reported substantially higher academic well-being (4.37 versus 3.57, *z* = 8.41, *p* < 0.001, *d* = 1.03) and academic motivation (3.90 versus 3.14, *z* = 7.25, *p* < 0.001, *d* = 0.96) and moderately lower parental achievement pressure (2.37 versus 2.90, *z* = −3.36, *p* = 0.002, *d* = −0.50) and self-handicapping (2.29 versus 2.57, *z* = −2.00, *p* = 0.046, *d* = −0.30). All four comparisons remained significant after Holm correction. Profile means (BCH-corrected) and distal outcome comparisons are shown in Table 7.

## 4. Discussion

This study investigated how the two dimensions of perfectionism relate to academic well-being in gifted adolescents and whether academic self-handicapping accounts for part of that relation. The clearest result is methodological before it is substantive, and study begins there since it conditions everything that follows. Academic motivation and subjective academic well-being were not empirically separable in this sample: their latent correlation was 0.955, and the separation did not improve when motivation was specified with the two factors the data actually supported. The motivational pathway we set out to test therefore could not be estimated as specified. We report a simple indirect model instead, and it is worth stating plainly that a substantial part of what the submitted version described as a mediational sequence was measurement overlap rather than a psychological process.

This has implications beyond the present paper. Academic motivation and academic well-being are routinely modelled as distinct constructs, often as sequential links in a causal chain, and the instruments used to measure them frequently share item content concerning interest, enjoyment and engagement with schoolwork. When two such measures are administered together in the same domain and at the same moment, a high correlation is not evidence of a strong effect; it may be evidence that the same thing has been measured twice. We would encourage researchers proposing motivation-to-well-being pathways to report a discriminant-validity test before interpreting the path coefficient, as we should have done at the outset.

What survives the correction is a coherent pattern organised around self-handicapping. Higher perfectionistic standards were associated with less self-handicapping and, through it, with higher academic well-being. Perceived parental achievement pressure showed the mirror-image pattern. Both indirect associations held under parcelled and item-level specification, under frequentist and Bayesian estimation, and both were statistically reliable with ample power. They were also small: most of their posterior mass fell inside a region we had defined in advance as practically negligible. We report them as reliable but modest, and we would not build a practical recommendation on associations of this magnitude.

The association between standards and better academic outcomes should not be read as evidence that high standards are protective, and we have removed that word. Perfectionistic strivings are frequently associated with positive academic outcomes, particularly once perfectionistic concerns are partialled out (Hill & Curran, 2016; Rice & Richardson, 2014), and our finding is consistent with the relevant literature and with Torok et al.’s (2022) demonstration that striving and concerns predict self-handicapping in opposite directions. But an association is not a benefit. There is a meaningful distinction between perfectionistic striving, which carries a self-evaluative edge, and a healthy pursuit of excellence, which does not, and our measure does not separate them. With cross-sectional data, the direction is unresolved in any case: students who already feel good about school may report higher standards as readily as high standards may accompany feeling good about school. We therefore confine the claim to the observation that higher standards were associated with less self-handicapping and higher academic well-being in this sample.

Discrepancy was not associated with self-handicapping, with academic well-being, or with parental pressure. This was contrary to expectation and deserves careful handling. Three readings are available. The first is substantive and contextual: in an academically selective setting, the felt gap between aspiration and attainment may be so widely shared that it ceases to differentiate students, which would explain why a dimension that predicts distress in unselected samples (Benedetto et al., 2024; Smith et al., 2018) does little work here. The second is psychometric: the subscale’s reliability was modest, one item loaded weakly, and the APS-R measurement model fitted poorly even after re-estimation. The third is that the null is genuine. Our evidence bears on the second and third. Removing the weakly loading item changed reliability trivially and left the associations unchanged, so the null is not an artefact of that item; and the Bayesian analysis placed 99.9% of the posterior for the indirect association inside the negligible region, which is positive evidence of a negligible association rather than mere failure to reject. Even so, we state only that discrepancy was not associated with the study variables in this sample. We have removed the words inert and carries little weight, and we make no claim that discrepancy is unimportant for gifted adolescents in general.

Parental achievement pressure was associated with more self-handicapping and, indirectly, with lower academic well-being. We no longer describe it as a source of self-handicapping or nominate it as the place to intervene, because the design licenses neither phrase. Students who handicap themselves, or who are struggling academically, may well elicit more pressure from parents, and the reverse ordering is equally consistent with these data. This is not a pedantic caveat: cross-sectional indirect associations can differ substantially in magnitude and even in sign from the longitudinal processes they are taken to represent and can appear when the corresponding longitudinal effect is absent (Maxwell & Cole, 2007; Maxwell et al., 2011). What we can say is that the pattern is consistent with work linking pressuring achievement climates to maladaptive coping among high-ability performers (Gustafsson et al., 2016) and with qualitative accounts in which parents and teachers in gifted settings describe competitive pressure and steep expectations as a strain on students (Noor, 2023).

The moderation hypothesis found no support, and here the distinction between absence of evidence and evidence of absence matters. The interaction interval included zero, but interaction effects require considerably more power than main effects, and we did not prespecify a smallest interaction effect of interest against which an equivalence test could have been conducted. We therefore report only that no evidence of moderation was found. Whether parental pressure conditions the cost of self-handicapping remains open and would be better addressed in a design powered for it.

Cultural context deserves more than a passing note. Parental achievement pressure in Turkiye takes shape within a highly competitive, examination-centred selection system in which placement at successive educational transitions carries substantial consequences and within family arrangements where academic attainment often carries collective rather than purely individual meaning. Behaviours the scale treats as pressure, such as comparison with peers or persistent dissatisfaction with results, may therefore be more normative here than in the settings where much of the perfectionism literature has been developed and may be interpreted differently by the students who experience them. This cuts both ways: it may attenuate associations if pressure is widely distributed, and it limits how far the present pattern should be generalised to other educational systems.

The person-centred analysis produced a two-profile solution separating students on standards alone; the profiles were indistinguishable on discrepancy. This is a more modest result than the three-profile solution reported in the submitted version, and the change followed directly from removing theoretical expectation from the enumeration criteria. It also answers a fair question about what such analyses contribute: in this case, very little beyond the standards dimension considered on its own. The adaptive, maladaptive and non-perfectionist configurations that the APS-R tradition anticipates, and which have been recovered in school samples elsewhere (Kuusi et al., 2024; Stahlberg et al., 2021), did not emerge here. Whether that reflects the restricted range of a selective sample, the modest measurement of discrepancy, or the use of only two profiling indicators, we cannot determine.

Several limitations bound these conclusions, and some are serious. The design is cross-sectional and drawn from a single site, so no temporal sequence can be established and every association reported is concurrent. All constructs were assessed by student self-report at one sitting, which inflates associations among conceptually adjacent measures, as the motivation and well-being result illustrates directly. The structural model, although better fitting than the one it replaces, still falls short of the Hu and Bentler benchmarks the paper adopts, and the APS-R measurement model fits poorly, so conclusions involving discrepancy are correspondingly weak. Parcelling was used in the primary model and can conceal multidimensionality and item-level misspecification (Little et al., 2013); we addressed this with a full item-level replication, but the general caution stands. Gender invariance rests on a configural baseline that does not itself fit well, so group comparisons should be treated as provisional. The sample was identified through procedures specific to the Turkish system, and the identification domain constrains generalisation. Finally, the reported indirect associations, while reliable, are small in absolute terms.

## 5. Conclusions

In gifted adolescents, high personal standards and low family pressure were associated with better academic well-being along a motivational route that ran partly through reduced self-handicapping, while the self-critical discrepancy facet added little. For teachers and counsellors, the takeaway is less about suppressing perfectionism wholesale than about nurturing healthy standards and easing pressuring family climates (Grugan et al., 2025).

## Figures and Tables

**Table 1:** Descriptive statistics, internal consistencies and correlations among variables.

Variable	*M*	*SD*	Omega	1	2	3	4	5	6
1. Subjective academic well-being	3.89	0.79	0.85	—					
2. Academic motivation	3.45	0.79	0.93	0.83 ***	—				
3. Academic self-handicapping	2.46	0.98	0.81	−0.45 ***	−0.40 ***	—			
4. Perfectionistic standards	5.32	1.30	0.84	0.68 ***	0.66 ***	−0.28 ***	—		
5. Perfectionistic discrepancy	3.72	1.30	0.64	0.03	0.07	0.05	0.12	—	
6. Parental achievement pressure	2.69	1.07	0.93	−0.27 ***	−0.18 **	0.31 ***	−0.19 **	0.02	—

Note. *N* = 234. ** *p* < 0.01. *** *p* < 0.001.

**Table 2:** Measurement model fit under WLSMV estimation.

Model	CFI	TLI	RMSEA	SRMR	chi2	df
Subjective academic well-being (1 factor)	0.988	0.980	0.090	0.044	26.0	9
Academic self-handicapping (1 factor)	0.949	0.915	0.145	0.063	53.0	9
Parental achievement pressure (1 factor)	0.945	0.936	0.132	0.076	448.4	90
Academic motivation (2 factors)	0.941	0.934	0.083	0.065	426.4	169
APS-R (1 factor)	0.711	0.595	0.293	0.161	413.8	20
APS-R (2 factors)	0.865	0.801	0.205	0.111	202.4	19

Note. *N* = 234.

**Table 3:** Discriminant validity models.

Model	CFI	TLI	RMSEA	SRMR	Latent *r* with Well-Being
One factor (motivation and well-being merged)	0.923	0.916	0.087	0.080	-
Two factors (motivation, well-being)	0.924	0.917	0.087	0.079	0.955
Three factors (M1, M2, well-being)	0.937	0.931	0.079	0.072	0.940/0.901

Note. *N* = 234; WLSMV estimation.

**Table 4:** Standardised structural coefficients for parcelled and item-level models.

Path	Parcelled Model	Item-Level Model
Discrepancy → Self-handicapping	0.068	NA
Standards → Self-handicapping	−0.295 **	−0.301 ***
Parental pressure → Self-handicapping	0.275 ***	0.324 ***
Self-handicapping → Academic well-being	−0.248 ***	−0.292 ***
Discrepancy → Academic well-being	−0.007	NA
Standards → Academic well-being	0.702 ***	0.664 ***
Parental pressure → Academic well-being	0.030	−0.038

Note. ** *p* < 0.01. *** *p* < 0.001.

**Table 5:** Indirect associations with Monte Carlo intervals and ROPE results.

Indirect Association	Estimate	95% MC CI	Posterior in ROPE (%)
Discrepancy → SH → Academic well-being	−0.013	[−0.051, 0.030]	99.9
Standards → SH → Academic well-being	0.053	[0.016, 0.102]	86.2
Parental pressure → SH → Academic well-being	−0.049	[−0.097, −0.015]	78.5

Note. SH = self-handicapping; ROPE defined as |beta| < 0.05.

**Table 6:** Latent profile enumeration.

Model	K	logLik	AIC	BIC	aBIC	Entropy
1class	1	−663.06	1332.12	1342.49	1332.98	-
EEI	2	−660.79	1335.59	1359.77	1337.59	0.375
EEI	3	−643.39	1306.79	1341.34	1309.65	0.570
EEI	4	−635.94	1297.87	1342.79	1301.59	0.675
EEI	5	−634.06	1300.12	1355.40	1304.69	0.704
VVI	2	−626.43	1270.85	1301.95	1273.43	0.712
VVI	3	−618.72	1265.45	1313.82	1269.45	0.742
VVI	4	−617.01	1272.01	1337.66	1277.44	0.703
VVI	5	−603.24	1254.49	1337.41	1261.34	0.871
EEE	2	−660.70	1337.40	1365.05	1339.69	0.371
EEE	3	−642.15	1306.30	1344.31	1309.45	0.591
EEE	4	−635.93	1299.86	1348.23	1303.86	0.671
EEE	5	−634.03	1302.06	1360.80	1306.92	0.703
VVV	2	−622.09	1266.19	1304.20	1269.33	0.732
VVV	5	−589.65	1237.29	1337.50	1245.58	0.850

Note. *N* = 234; indicators standardised. Solutions that failed to converge are omitted.

**Table 7:** Profile means (BCH-corrected) and distal outcome comparisons.

Variable	Profile 1 (*n* = 85)	Profile 2 (*n* = 149)	*z*	*p*	*d*
Perfectionistic standards	6.79 (0.04)	4.33 (0.10)	22.63	0.000	1.89
Discrepancy	3.77 (0.18)	3.69 (0.11)	0.36	0.715	0.06
Academic well-being	4.37 (0.06)	3.57 (0.08)	8.41	0.000	1.03
Academic motivation	3.90 (0.08)	3.14 (0.07)	7.25	0.000	0.96
Self-handicapping	2.29 (0.11)	2.57 (0.09)	−2.00	0.046	−0.30
Parental achievement pressure	2.37 (0.13)	2.90 (0.09)	−3.36	0.001	−0.50

Note. Standard errors in parentheses; Holm-corrected *p* values.

## Data Availability

The data that support the findings of this study are available on request from the author. The data are not publicly available due to privacy or ethical restrictions.

## References

[B1-behavsci-16-01904] Anlı G., Taş İ., Güneş Z., Yazgı Z., Sevinç H. (2018). Akademik kendini engelleme ölçeği’ni Türkçeye uyarlama çalişmasi. OPUS International Journal of Society Researches.

[B2-behavsci-16-01904] Arslan G., Yıldırım M., Albertova S. M. (2022). Development and initial validation of the subjective academic wellbeing measure: A new tool of youth wellbeing in school. Journal of Positive School Psychology.

[B3-behavsci-16-01904] Asparouhov T., Muthen B. (2014). Auxiliary variables in mixture modeling: Three-step approaches using Mplus. Structural Equation Modeling.

[B4-behavsci-16-01904] Bakker A. B., Mostert K. (2024). Study demands-resources theory: Understanding student well-being in higher education. Educational Psychology Review.

[B5-behavsci-16-01904] Benedetto L., Macidonio S., Ingrassia M. (2024). Well-being and perfectionism: Assessing the mediational role of self-compassion in emerging adults. European Journal of Investigation in Health, Psychology and Education.

[B6-behavsci-16-01904] Bolck A., Croon M., Hagenaars J. (2004). Estimating latent structure models with categorical variables: One-step versus three-step estimators. Political Analysis.

[B7-behavsci-16-01904] Bozanoglu I. (2004). Akademik Gudulenme Olcegi: Gelistirmesi, gecerligi, guvenirligi. Ankara University Journal of Faculty of Educational Sciences.

[B8-behavsci-16-01904] Bureau J. S., Howard J. L., Chong J. X. Y., Guay F. (2022). Pathways to student motivation: A meta-analysis of antecedents of autonomous and controlled motivations. Review of Educational Research.

[B9-behavsci-16-01904] Casino-Garcia A. M., Garcia-Perez J., Llinares-Insa L. I. (2019). Subjective emotional well-being, emotional intelligence, and mood of gifted vs. unidentified students: A relationship model. International Journal of Environmental Research and Public Health.

[B10-behavsci-16-01904] Chen F. F. (2007). Sensitivity of goodness of fit indexes to lack of measurement invariance. Structural Equation Modeling: A Multidisciplinary Journal.

[B11-behavsci-16-01904] Curran T., Hill A. P. (2019). Perfectionism is increasing over time: A meta-analysis of birth cohort differences from 1989 to 2016. Psychological Bulletin.

[B12-behavsci-16-01904] Findik H., Afat N. (2023). Perfectionism and life satisfaction in gifted students. International Journal of Psychology and Educational Studies.

[B13-behavsci-16-01904] Flora D. B., Curran P. J. (2004). An empirical evaluation of alternative methods of estimation for confirmatory factor analysis with ordinal data. Psychological Methods.

[B14-behavsci-16-01904] Fox J., Weisberg S. (2019). An R companion to applied regression.

[B15-behavsci-16-01904] Grugan M. C., Olsson L. F., Hill A. P., Madigan D. J. (2025). Perfectionism, school burnout, and school engagement in gifted students: The role of stress. Gifted Child Quarterly.

[B16-behavsci-16-01904] Guo J., Gabry J., Goodrich B., Johnson A., Weber S., Badr H. S. (2015). rstan: R interface to Stan *(R package Version 2.32.7) [Computer software]*.

[B17-behavsci-16-01904] Gustafsson H., Hill A. P., Stenling A., Wagnsson S. (2016). Profiles of perfectionism, parental climate, and burnout among competitive junior athletes. Scandinavian Journal of Medicine & Science in Sports.

[B18-behavsci-16-01904] Haspolat N. K., Yalcin I. (2022). Yuksek basarili ogrencilerde ebeveyn basari baskisi olcegi gelistirme calismasi. Milli Egitim Dergisi.

[B19-behavsci-16-01904] Hill A. P., Curran T. (2016). Multidimensional perfectionism and burnout: A meta-analysis. Personality and Social Psychology Review.

[B20-behavsci-16-01904] Hill A. P., Madigan D. J. (2022). Perfectionism, mattering, stress, and self-regulation of home learning of UK gifted and talented students during the COVID-19 pandemic. Gifted and Talented International.

[B21-behavsci-16-01904] Hothorn T., Zeileis A., Farebrother R. W., Cummins C. (1999). lmtest: Testing linear regression models *(R package Version 0.9-40) [Computer software]*.

[B22-behavsci-16-01904] Hu L., Bentler P. M. (1999). Cutoff criteria for fit indexes in covariance structure analysis: Conventional criteria versus new alternatives. Structural Equation Modeling.

[B23-behavsci-16-01904] Jorgensen T. D., Pornprasertmanit S., Schoemann A. M., Rosseel Y. (2012). semTools: Useful tools for structural equation modeling *(R package Version 0.5-7) [Computer software]*.

[B24-behavsci-16-01904] Kara E., Aslan H. (2025). Cyber victimization and subjective well-being: Protective roles of self-esteem and social support among Turkish gifted youths. BMC Psychology.

[B25-behavsci-16-01904] Kokcam B., Tras Z. (2026). Ergenlerde APS-R Mukemmeliyetcilik Olcegi Turkce Kisa Formunun gecerlik ve guvenirliginin incelenmesi. Turk Egitim Bilimleri Dergisi.

[B26-behavsci-16-01904] Korkmaz S., Goksuluk D., Zararsiz G. (2014). MVN: An R package for assessing multivariate normality. The R Journal.

[B27-behavsci-16-01904] Kruschke J. K. (2018). Rejecting or accepting parameter values in Bayesian estimation. Advances in Methods and Practices in Psychological Science.

[B28-behavsci-16-01904] Kuusi A., Tuominen H., Widlund A., Korhonen J., Niemivirta M. (2024). Lower secondary students’ perfectionistic profiles: Stability, transitions, and connections with well-being. Learning and Individual Differences.

[B29-behavsci-16-01904] Little T. D., Rhemtulla M., Gibson K., Schoemann A. M. (2013). Why the items versus parcels controversy needn’t be one. Psychological Methods.

[B30-behavsci-16-01904] Maxwell S. E., Cole D. A. (2007). Bias in cross-sectional analyses of longitudinal mediation. Psychological Methods.

[B31-behavsci-16-01904] Maxwell S. E., Cole D. A., Mitchell M. A. (2011). Bias in cross-sectional analyses of longitudinal mediation: Partial and complete mediation under an autoregressive model. Multivariate Behavioral Research.

[B32-behavsci-16-01904] McCrea S. M. (2008). Self-handicapping, excuse making, and counterfactual thinking: Consequences for self-esteem and future motivation. Journal of Personality and Social Psychology.

[B33-behavsci-16-01904] Merkle E. C., Fitzsimmons E., Uanhoro J., Goodrich B. (2021). Efficient Bayesian structural equation modeling in Stan. Journal of Statistical Software.

[B34-behavsci-16-01904] Merkle E. C., Rosseel Y. (2018). blavaan: Bayesian structural equation models via parameter expansion. Journal of Statistical Software.

[B35-behavsci-16-01904] Noor B. (2023). Pressure and perfectionism: A phenomenological study on parents’ and teachers’ perceptions of the challenges faced by gifted and talented students in self-contained classes. Frontiers in Education.

[B36-behavsci-16-01904] Nylund-Gibson K., Choi A. Y. (2018). Ten frequently asked questions about latent class analysis. Translational Issues in Psychological Science.

[B37-behavsci-16-01904] Preacher K. J. (2015). Advances in mediation analysis: A survey and synthesis of new developments. Annual Review of Psychology.

[B38-behavsci-16-01904] Raoof K., Shokri O., Fathabadi J., Panaghi L. (2024). Unpacking the underachievement of gifted students: A systematic review of internal and external factors. Heliyon.

[B39-behavsci-16-01904] R Core Team (2025). R: A language and environment for statistical computing *(Version 4.5.2) [Computer software]*.

[B40-behavsci-16-01904] Revelle W. (2007). psych: Procedures for psychological, psychometric, and personality research *(R package Version 2.5.6) [Computer software]*.

[B41-behavsci-16-01904] Rhemtulla M., Brosseau-Liard P. E., Savalei V. (2012). When can categorical variables be treated as continuous?. Psychological Methods.

[B42-behavsci-16-01904] Rice K. G., Richardson C. M. E. (2014). Classification challenges in perfectionism. Journal of Counseling Psychology.

[B43-behavsci-16-01904] Rice K. G., Richardson C. M. E., Tueller S. (2014). The short form of the revised almost perfect scale. Journal of Personality Assessment.

[B44-behavsci-16-01904] Rosenberg J. M., Beymer P. N., Anderson D. J., Van Lissa C. J., Schmidt J. A. (2018). tidyLPA: An R package to easily carry out latent profile analysis using open-source or commercial software. Journal of Open Source Software.

[B45-behavsci-16-01904] Rosseel Y. (2012). lavaan: An R package for structural equation modeling. Journal of Statistical Software.

[B46-behavsci-16-01904] Scrucca L., Fraley C., Murphy T. B., Raftery A. E. (2023). Model-based clustering, classification, and density estimation using mclust in R.

[B47-behavsci-16-01904] Smith M. M., Sherry S. B., Chen S., Saklofske D. H., Mushquash C., Flett G. L., Hewitt P. L. (2018). The perniciousness of perfectionism: A meta-analytic review of the perfectionism-suicide relationship. Journal of Personality.

[B48-behavsci-16-01904] Stahlberg J., Tuominen H., Pulkka A.-T., Niemivirta M. (2021). Students’ perfectionistic profiles: Stability, change, and associations with achievement goal orientations. Psychology in the Schools.

[B49-behavsci-16-01904] Thomas C. R., Gadbois S. A. (2006). Academic self-handicapping: The role of self-concept clarity and students’ learning strategies. British Journal of Educational Psychology.

[B50-behavsci-16-01904] Tierney N., Cook D. (2023). Expanding tidy data principles to facilitate missing data exploration, visualization and assessment of imputations. Journal of Statistical Software.

[B51-behavsci-16-01904] Torok L., Szabo Z. P., Orosz G. (2022). Elite athletes’ perfectionistic striving vs. concerns as opposing predictors of self-handicapping with the mediating role of attributional style. Frontiers in Psychology.

[B52-behavsci-16-01904] Urdan T., Midgley C. (2001). Academic self-handicapping: What we know, what more there is to learn. Educational Psychology Review.

[B53-behavsci-16-01904] Vansteenkiste M., Ryan R. M. (2013). On psychological growth and vulnerability: Basic psychological need satisfaction and need frustration as a unifying principle. Journal of Psychotherapy Integration.

[B54-behavsci-16-01904] Wickham H. (2016). ggplot2: Elegant graphics for data analysis.

